# Oral cancer awareness campaign in Northern Germany: first positive trends in incidence and tumour stages

**DOI:** 10.1007/s00432-020-03305-8

**Published:** 2020-07-02

**Authors:** Katrin Hertrampf, Ron Pritzkuleit, Eva Baumann, Jörg Wiltfang, Hans-Jürgen Wenz, Annika Waldmann

**Affiliations:** 1 Clinic of Oral and Maxillofacial Surgery, University Hospital of Schleswig-Holstein, Campus Kiel, Arnold-Heller-Str. 3, Building B, 24105 Kiel, Germany; 2 Institute for Cancer Epidemiology, University of Lübeck, Ratzeburger Allee 160, 23562 Lübeck, Germany; 3 Department of Journalism and Communication Research, Hannover University of Music, Drama, and Media, Expo Plaza 12, 30539 Hannover, Germany; 4 Clinic of Prosthodontics, Propaedeutics and Dental Materials, University Hospital of Schleswig-Holstein, Campus Kiel, Arnold-Heller-Str.3, Building B, 24105 Kiel, Germany; 5 Institute of Social Medicine and Epidemiology, University of Lübeck, Ratzeburger Allee 160, 23562 Lübeck, Germany

**Keywords:** Incidence, Tumour stages, Oral cancer, Awareness campaign, Germany

## Abstract

**Purpose:**

Oral cancer is a still underestimated public health problem. In Germany, until 2007, there was no systematic approach available for the purpose of raising the awareness of the public. From 2007 to 2011, a concept was developed for such an approach, and the campaign was launched in Northern Germany in 2012, and concluded in 2014. This report aims at presenting incidence trends of oral cancer, stratified according to sex, age, and tumour stages, before the introduction of this campaign and upon completion thereof.

**Methods:**

The data kept by the Schleswig–Holstein Cancer Registry on incidence rates (ICD-10, C00–C14) focused on oral cancer (C00–C06) and stratified by sex, age-groups and tumour stages, from 2000 to 2006 and from 2007 to 2014.

**Results:**

From 2000 to 2014, a total of 6760 cases of oral and pharyngeal cancer (C00–C14) were registered. When data on oral cancer was taken into account, stage I cancers of women in particular, increased over time. Regarding the stages, stage IV was the most frequent and stage I the second most frequent stage for both men and women. Over time, a small shift towards detection of tumours at earlier stages was observed.

**Conclusion:**

A slight trend towards a temporary increase in incidence rates, especially among women, was observed. From an epidemiological point of view, this might indicate the initial success of this campaign. The slight trend in favour of stage I tumours could be seen as an initial minor success in terms of the early detection of oral cancer.

## Introduction

Oral and pharyngeal cancer is a serious, but still underestimated public health problem. The global incidence rates for this combined tumour group (oral cavity including lips, pharyngeal region, ICD-10 C00–C14), were estimated at more than 529,000 newly diagnosed cases, which corresponds to 3.8% of all cancers in 2012 (Shield et al. 2017). In Germany, the incidence rates of oral (including lips) and pharyngeal cancer has increased over the past years, from 10,000 to more than 14,000 newly diagnosed cases per year, and it currently rates in 9th place (3.7% of all malignancies) amongst men and 15th place (1.6% amongst women), (Robert Koch-Institut and GdeKiD eV 2019). The majority of patients with oral and pharyngeal cancer only consult a doctor or dentist when the tumour is at an advanced stage. These late stages are more likely to require extensive therapy, which entails an impaired quality of life and a significantly unfavourable prognosis for the patients concerned. To date, the 5-year survival rate in Germany is rather low (47% for men and 63% for women), (Robert Koch-Institut and GdeKiD eV 2019; Sankaranarayanan et al. 2013).

With a focus on oral cancer, a variety of international studies—including the studies of the authors—have shown that the knowledge of the general public regarding signs and symptoms, and the available measures of prevention and early detection, especially for this tumour site, tend to be very insufficient (Hertrampf et al. 2012a; Jedele and Ismail 2010; Logan et al. 2013).

Furthermore, only very few systematic prevention campaigns have been carried out for raising awareness about oral cancer (Eadie et al. 2009; Jedele and Ismail 2010; Logan et al. 2013; Watson et al. 2009).

In Germany, until 2007, no comprehensive and systematic approach was available for the purpose of raising the awareness of the public or different risk groups, or on how to provide information about oral cancer and preventive measures for the disease. In that year, a subgroup of the authors undertook a comprehensive assessment of the required elements for the development and assessment of an awareness campaign themselves. Four subsections were identified: (i) media coverage/mass media, (ii) target groups, (iii) dental and medical healthcare professionals, and (iv) the epidemiological data. Subsequently, corresponding work packages were developed and implemented. For more details on the development and assessment of the campaign, with regard to subsections (i)–(iii), see (Baumann et al. 2019).

For the epidemiological data (iv), a detailed analysis of baseline trends in incidence and mortality rates was conducted. As the campaign focused exclusively on oral cancer, the analyses were not only stratified according to age and gender, but also according to the tumour site; and furthermore, according to tumour stages (Hertrampf et al. b, c). Concerning the mass media (i), an oral cancer awareness campaign was launched in Northern Germany from April 2012 to December 2014. It was the first federal state-wide campaign to be conducted (Baumann et al. 2019).

The evaluation of epidemiological data from population-based cancer registries (see iv) is an essential component in terms of the success of an awareness campaign with oncological focus (White et al. 2017; Wingo et al. 2005). Subsequently to such an awareness campaign, one would expect a possible temporary increase (a so-called “prevalence peak”) and furthermore, also a shift of the tumour stages towards earlier stages.

In this report, we, therefore, aimed at presenting incidence trends for oral cancer, stratified according to gender, age, tumour sub-site and tumour stages, before the introduction and after the conclusion of the federal state-wide oral cancer awareness campaign in Schleswig–Holstein, Northern Germany.

## Material and methods

### Epidemiological data

The incidence data on oral and pharyngeal cancer (ICD-10 C00–C14) for the period 2000 to 2014 was provided by the Cancer Registry of the Federal State of Schleswig–Holstein. In accordance with the mandatory notification required by federal state law, all newly diagnosed tumour cases within the population of Schleswig–Holstein (approx. 2.8 million inhabitants; reference: Federal Statistical Office Germany) have been registered throughout the federal state since 1998. All tumour diagnoses were encoded according to the International Classification of Diseases (ICD-10): oral cancer (C00–C06), salivary glands (C07–C08), and pharynx (C09–C14). Morphology was described by the International Classification of Diseases for Oncology (ICD-O-3). The evaluation of the tumour stages, lymph nodes and metastases followed the TMN Classification of the Union Internationale Contre le Cancer (until the year 2010: UICC, 6th Edition, from 2011 on: UICC 7th Edition).

Incidence rates were provided as age-standardised rates (previously European standard population; ASR [E]) per 100,000 persons (Waterhouse et al. 1976) and were stratified according to sex and sub-site. Age-specific rates for women and men were presented in the following age groups: 0–39, 40–59, 60–79, 80 years or older.

As the federal state-wide campaign focused on oral cancer, the stratified analyses, as described, focused on this tumour site. Parts of the results were presented within two different timelines, from 2000 to 2006, and from 2007 to 2014, i.e. the periods before and subsequent to the awareness campaign.

### Awareness campaign

An important aspect of the development of the campaign was the early and constructive involvement of the dental and medical health care professionals (iii). Therefore, a 1-year work package targeting this group was developed and implemented before the campaign was launched in April 2012. Based on this programme, beginning in autumn 2007, dental and medical healthcare professionals were invited to participate in the surveys. They were involved in different modes and for various durations, and they took part in further education programmes and finally, they supported the campaign. The whole concept for the healthcare professional was published separately (Baumann et al. 2019; Hertrampf et al. 2011, 2013).

### Ethics

Ethical approval was not required, as we used administrative data that did not allow for identification of individual persons.

## Results

From 2000 to 2014, a total of 6,760 cases (4787 men and 1973 women) of oral and pharyngeal cancer (C00–C14) were registered by the cancer registry of Schleswig–Holstein. An overview of the epidemiological data for the whole study period and a comparison between the years 2000–2006 and 2007–2014 is shown in Table 1.

**Table 1:** Descriptive results for oral and pharyngeal cancer 2000–2014, T1–4, Tx, in Schleswig–Holstein, Germany

	2000–2006				2007–2014				2000–2014			
	Incidence		Mortality		Incidence		Mortality		Incidence		Mortality	
	Men	Women	Men	Women	Men	Women	Men	Women	Men	Women	Men	Women
Oral and pharyngeal cancer (ICD-10 C00–C14)												
Number of cases	2115	834	807	277	2672	1139	875	341	4787	1973	1682	618
Crude rate	22.0	8.3	8.4	2.8	24.2	9.9	7.9	3.0	23.2	9.1	8.1	2.9
ASR [E]^a^	18.6	6.3	6.9	1.8	18.2	7.0	5.7	1.8	18.2	6.6	6.2	1.8
Age (median value) for 2014	–	–	–	–	–	–	–	–	65	65	69	71
Histology^c^ (in %)												
Squamous cell carcinoma	90.4	81.9	–	–	91.1	85.5	–	–	90.8	84.0	–	–
Adenocarcinoma	4.3	10.6	–	–	4.9	9.5	–	–	4.6	9.9	–	–
Sarcoma and other tumours of the soft tissue	0.2	0.6	–	–	0.1	0.4	–	–	0.2	0.5	–	–
Other carcinoma	4.2	6.4	–	–	3.1	3.6	–	–	3.6	4.8	–	–
Other tumours	0.9	0.6	–	–	0.8	1.1	–	–	0.9	0.9	–	–
Stratified by localisation/diagnosis group (ICD-10)^b^												
Oral cancer (C00–C06)												
Number of cases	1036	482	376	139	1265	623	339	167	2301	1105	715	306
Crude rate	10.8	4.8	3.9	1.4	11.5	5.4	3.1	1.4	11.1	5.1	3.5	1.4
ASR [E]^a^	9.1	3.5	3.3	0.9	8.6	3.7	2.2	0.8	8.7	3.6	2.6	0.9
Salivary glands (C07/08)												
Number of cases	132	96	44	42	154	91	42	27	286	187	86	69
Crude rate	1.4	1.0	0.5	0.4	1.4	0.8	0.4	0.2	1.4	0.9	0.4	0.3
ASR [E]^a^	1.0	0.7	0.3	0.2	1.0	0.5	0.3	0.2	1.0	0.6	0.3	0.2
Pharyngeal cancer (C09–C13)												
Number of cases	911	256	339	78	1230	415	454	135	2141	671	793	213
Crude rate	9.5	2.5	3.5	0.8	11.1	3.6	4.1	1.2	10.4	3.1	3.8	1.0
ASR [E]^a^	8.0	2.1	2.9	0.5	8.5	2.7	2.9	0.8	8.2	2.4	3.0	0.7
Others (C14)												
Number of cases	36	8	48	18	23	2	40	12	59	10	88	30
Crude rate	0.4	0.1	0.5	0.2	0.2	< 0.1	0.4	0.1	0.3	< 0.1	0.4	0.1
ASR [E]^a^	0.3	0.1	0.4	0.1	0.2	< 0.1	0.3	0.1	0.2	< 0.1	0.3	0.1

Data source: Cancer Registry Schleswig–Holstein

^a^Age-standardised rates (Europe ASR [E])

^b^International Classification of Diseases, ICD-10)

^c^International Classification of Diseases in Oncology, ICD-O-3)

The median age at diagnosis was 65 years. In both men and women, about 50% of the registered cases were between 60 and 79 years at the time of diagnosis, followed by cases in the age group from 40 to 59 years. About 91% of all cases of all oral and pharyngeal cancers in men and 84% in women were diagnosed as squamous cell carcinomas.

**Fig. 1:**
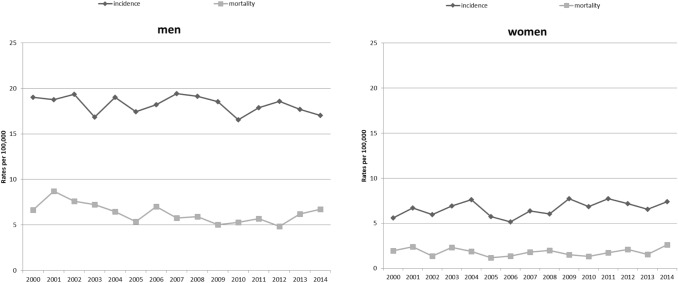
Incidence and mortality trends (ASR [E]) for oral and pharyngeal cancer (ICD-10 C00–C14) in men and women in Schleswig–Holstein from 2000 to 2014

The age-standardised rates (ASR [E]) of incidence for oral and pharyngeal cancer (C00–C14) in Schleswig–Holstein, stratified according to sex, are shown in Fig. 1. There was no obvious increase or decline in the trends regarding the incidence and the mortality rate over time.

**Fig. 2:**
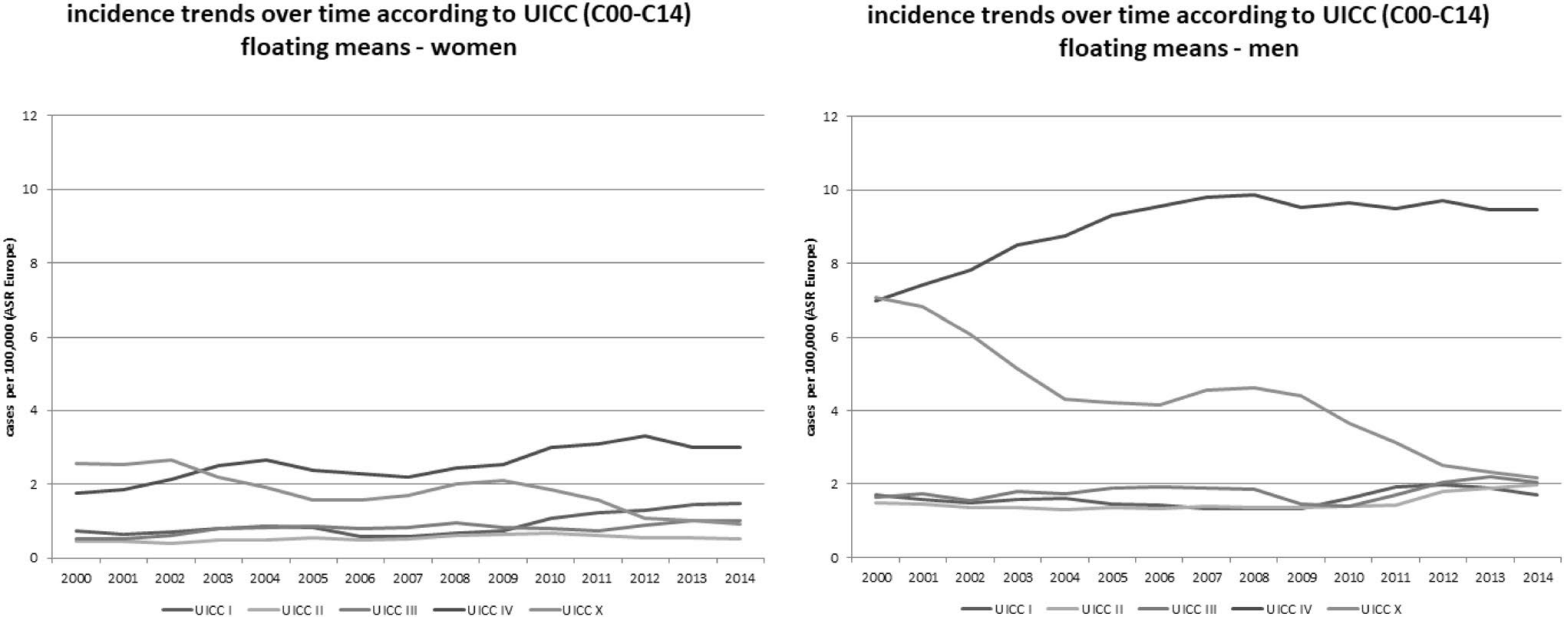
Incidence trends (ASR [E]) for different tumour stages (UICC; tumour stage I to IV; X = not known) for oral and pharyngeal cancer (ICD-10 C00–C14) in men and women in Schleswig–Holstein from 2000 to 2014

In Fig. 2, the incidence trends for different tumour stages (UICC) are shown, stratified according to sex. In men, the frequency of tumors with unknown stages (denoted as X) and stage IV were very similar in 2000, but over time, the incidence of stage IV tumours increased, while the incidence decreased for tumours with unknown stages. In women, the tumours of unknown stages and of stage IV were the most frequent tumours until 2010. Thereafter, a decrease was observed for unknown stages, but not for stage IV tumours. In both men and women, a slight increase was observed in the incidence of stages I–III.

**Fig. 3:**
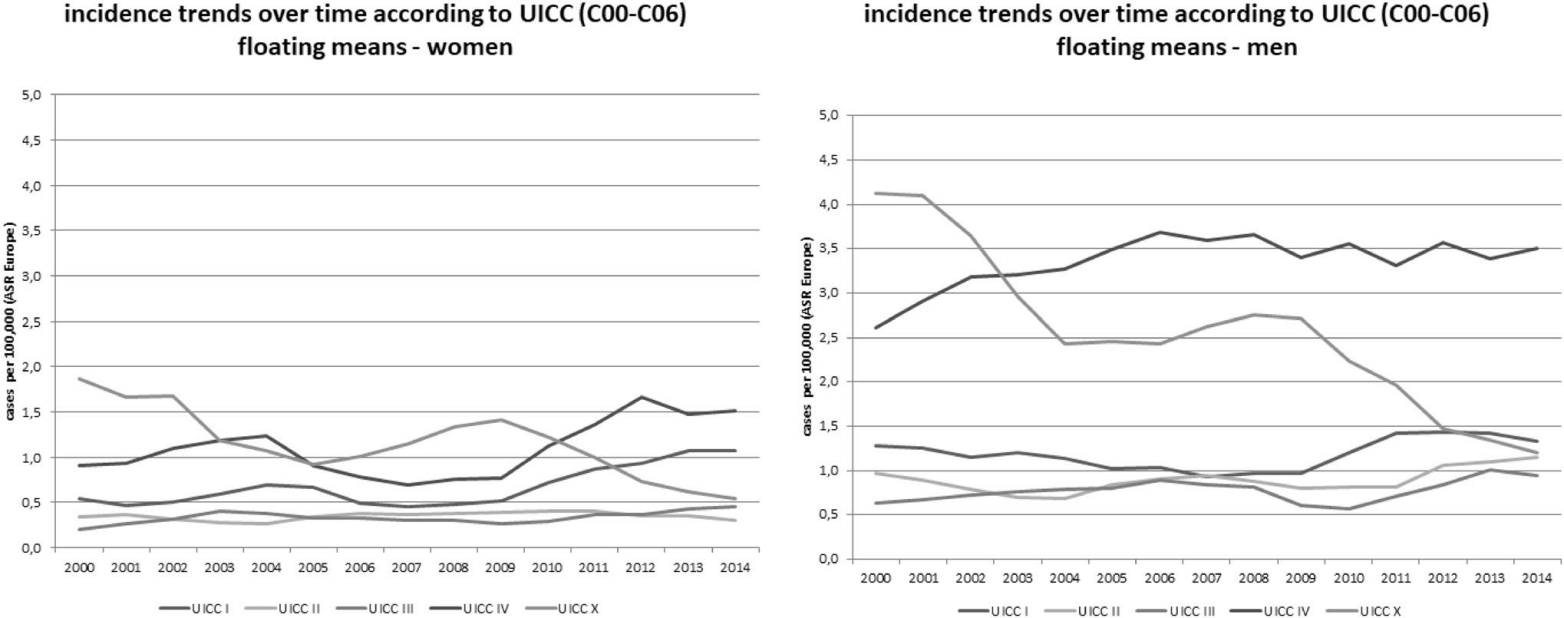
Incidence trends (ASR [E]) for different tumour stages (UICC; x = not known) for oral cancer (ICD-10 C00–C06) by tumour sites in men and women in Schleswig–Holstein from 2000 to 2014

When data on oral cancer (C00–C06)—which was the main target of the intervention—was focused, as shown in Fig. 3**,** stage I cancers in women in particular, increased over time.

**Fig. 4:**
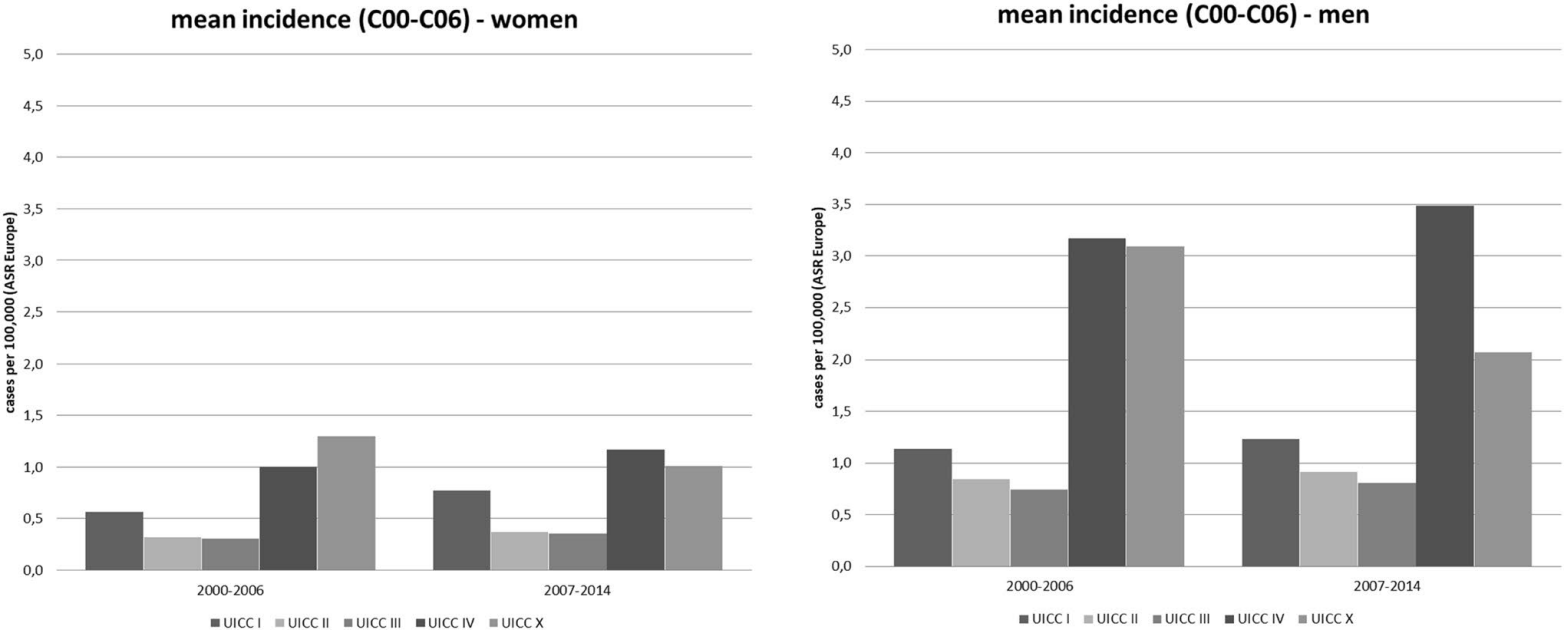
Different tumour stages (UICC) for oral cancer (ICD-10 C00–C06) stratified by time periods in men and women in Schleswig–Holstein

Absolute frequencies of tumour stages for oral cancer, diagnosed between 2000 and 2014, and further stratified according to time periods, are shown in Fig. 4. Of all cases at known stages, stage IV was the most frequent and stage I the second most frequent stage both for men and women. In women, the frequency of stage I tumours was nearly twice as high as that of stage II and of stage III cancers, respectively. Although over time, a small shift towards earlier tumour stages was observed, the change in the relative frequencies of the different tumour stages was not clinically relevant in men (UICC I: early period 20.0% vs. late period 19.4%; UICC IV: early period 53.6% vs. late period 53.8%) and women (UICC I: early period: 25.3% vs. late period: 28.9%, UICC IV: early period: 46.7% vs. late period 43.0%). In general, women were more likely to present with earlier tumor stages at primary diagnosis than men.

**Fig. 5:**
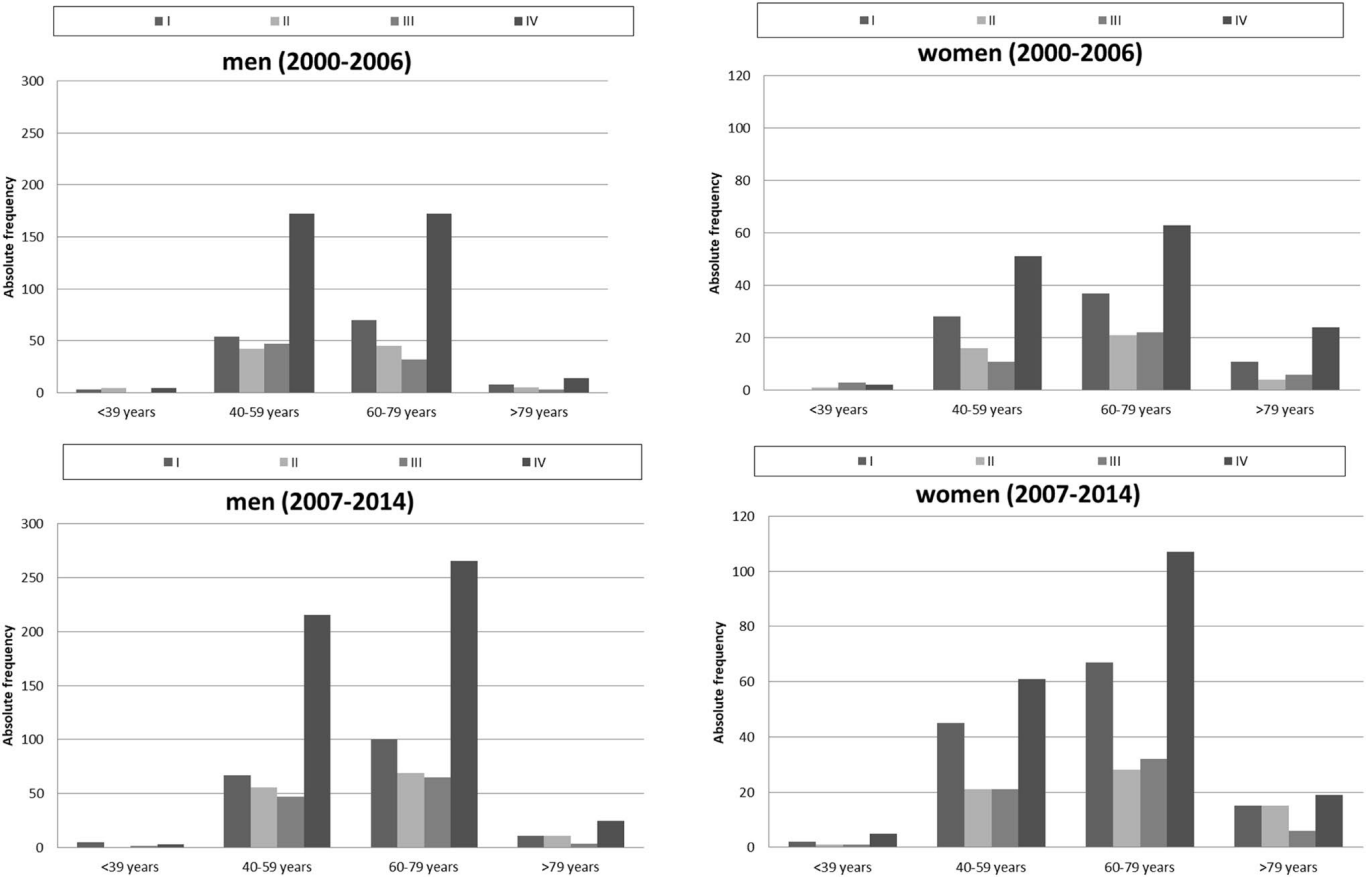
Trends for different tumour stages (UICC) for oral cancer (ICD-10 C00–C06) in men and women stratified by age groups and further by time periods (2000–2006; 2007–2014) in Schleswig–Holstein

The further stratification according to age groups demonstrated the same trends for tumour stage IV, but also the same small shift towards earlier tumour stages (Fig. 5).

## Discussion

This paper describes trends in incidence, tumour sites and tumour stages, according to age and sex, specifically for oral cancer, with regard to possible changes, based on the first federal state-wide oral cancer awareness campaign in Northern Germany.

The presentation of the results and the interpretation thereof deliberately takes into account the period before the involvement of the healthcare professionals through assessment, educational training, and evaluation (Hertrampf et al. 2011, 2013) and subsequent to the implementation of the campaign, and assessment after the conclusion of the campaign (2007–2014). For this reason, the following section will critically reflect on the results of the campaign, with regard to a temporary increase of incidence rates and/or a shift of tumour diagnosis stages towards earlier stages. We will also discuss the results in a European context.

In an earlier report, we demonstrated that in the combined group of ‘oral and pharyngeal cancer’ (ICD-10 C00–C14), the incidences in both sexes in Schleswig–Holstein, Northern Germany, in the period from 2000 to 2006, showed no clear upward or downward trend (Hertrampf et al. 2012a). In the current results, slightly increasing incidence rates were observed in women for the later period (i.e. 2007–2014). Incidence rates in men were more likely to show stagnation. When comparing the regional results in both sexes for this period, using the German estimates, the regional incidences for men were roughly in line with the national average. For women, however, it turned out that the rising incidences in Schleswig–Holstein were well above the national average, and furthermore, that they were among the highest in Germany (GeKiD 2019).

When comparing our results with the European average in 2012 and in 2015 (Ferlay et al. 2018, 2013), incidence rates for women in Schleswig–Holstein were well above the European average.

When stratifying according to tumour site and focusing on 'oral cancer' (ICD-10 C00–C06), which is the tumour site targeted by the awareness campaign, the incidence rate in women showed a small, but increasing trend in Schleswig–Holstein over time. This trend was also observed in the evaluation of the German estimates for the period from 2003 to 2011 (Hertrampf et al. 2015). Compared to the time period 2000–2006, the age-standardised incidence rates for 2007–2014 increased by 0.69 cases in women and by 0.75 cases in men, to 3.56 (women) and 9.23 (men) per 100,000.

In the European context, a heterogeneous picture of incidence rates for oral cancer (ICD-10 C00–C06) from 2000 to 2006 emerged. Germany was described as having intermediate rates of oral cancer compared to countries with higher incidence rates, such as Spain, Portugal and Switzerland, and countries with lower incidence rates, such as Greece, Finland and Sweden (Warnakulasuriya 2009). When sex was taken into account, a similar mixed picture emerged for this observation period. For example, while increasing trends were observed for men in France, increasing incidence rates for women were reported for Nordic countries (Curado and Hashibe 2009; Warnakulasuriya 2009). In a European comparison, based on the Globocan estimates for 2018, this heterogenous pattern is also evident: highest incidence rates were reported for men in Central and Eastern Europe and for women in Western and Northern Europe (Bray et al. 2018). However, compared to our own regional data, the regional incidence rates for men and women were higher than those recorded in the European data.

Another potential indicator for the assessment of an awareness campaign, from an epidemiological point of view, is a shift of the tumour diagnosis stages towards earlier stages, as the aim of this campaign was to inform people about the existence, the signs and symptoms, and the early detection of oral cancer.

In the period from 2000 to 2006, an earlier report based on data from Schleswig–Holstein showed that the majority of those affected with oral cancer were diagnosed at tumour stage IV (UICC), followed, at a very clear distance, by tumour stage I (Hertrampf et al. 2012b).

In our recent report, we observed that the majority of patients are still diagnosed at stage IV (Hertrampf et al. 2012b). However, it was found that the proportion of women diagnosed with stage I increased significantly from 2007 to 2014. In this respect, we would suggest a slight trend towards a shift to the left. Against the background of the rising incidence rates among women, this could be interpreted as the first success of the awareness campaign.

Unfortunately, a comparison of our site-specific results with data from other countries was not possible, due to the lack of published data on site-specific tumour stages. However, authors like Pulte and Brenner (2010) and de Camargo et al. (2010) have recommended these epidemiological assessments, especially with regard to the discussion of tumour stages, as they consider it an important aspect in the evaluation of cancer control measures (de Camargo et al. 2010; Pulte and Brenner 2010).

### Strengths and limitations

The main strength of this analysis is the use of high-quality data from a population-based cancer register. The Schleswig–Holstein Cancer Registry has been operational since 1998. The comprehensiveness of the diagnosed cases is considered to be high (more than 95%) (Robert Koch-Institut 2013), and the case ascertainment is almost 100%.

Furthermore, we were able to analyse sub-group specific incidence, while traditionally, data on oral and pharyngeal cancer from population-based cancer registries has been analysed and described for the total group ICD-10 C00–C14. This is especially important for monitoring the detailed effects of the national prevention campaign on incidence and mortality rates of oral cancer on the population-level (White et al. 2017).

Since this assessment focuses on trends in incidence rates and tumour stages in comparison with the baseline data before the implementation of the educational programme for healthcare professionals and the awareness campaign, we decided to focus on trend analysis, and to refrain from continuative statistical analysis.

## Conclusion

The oral cancer awareness campaign described here was conducted in the federal state of Schleswig–Holstein, Northern Germany. It was the first campaign of its kind to be conducted in Germany. The epidemiological assessment was an important part of an assessment concept to evaluate the success of a campaign with regard to indicators, such as a temporary increase in incidence rates and a shift of tumour diagnosis stages towards earlier stages.

Against the background of the slight overall increase in incidence rates for women in Germany, and also internationally, a trend towards a temporary increase in incidence rates, especially among women, was identified in the region affected by the study. From an epidemiological point of view, this could certainly be seen as an initial positive success for this campaign. In terms of an anticipated improved survival prognosis for these patients, the positive trend in favour of tumour stage I could also be seen as a success in terms of the early detection of oral cancer.

However, this comprehensive assessment also underlines the importance of integrating stratified, site-specific epidemiological assessment concepts into awareness campaigns, right from the start.
